# Quantitative trait locus analysis of agronomic and quality-related traits in cultivated peanut (*Arachis hypogaea* L.)

**DOI:** 10.1007/s00122-015-2493-1

**Published:** 2015-03-25

**Authors:** Li Huang, Haiyan He, Weigang Chen, Xiaoping Ren, Yuning Chen, Xiaojing Zhou, Youlin Xia, Xiaolin Wang, Xiangguo Jiang, Boshou Liao, Huifang Jiang

**Affiliations:** 1 Key Laboratory of Biology and Genetic Improvement of Oil Crops, Ministry of Agriculture, Oil Crops Research Institute of the Chinese Academy of Agricultural Sciences, Wuhan, 430062 China; 2 Nanchong Academy of Agricultural Sciences, Nanchong, 637000 China; 3 Zhumadian Academy of Agricultural Sciences, Zhumadian, 463000 China; 4 Xiangyang Academy of Agricultural Sciences, Xiangyang, 461057 China

## Abstract

*****Key message***:**

**SSR-based QTL mapping provides useful information for map-based cloning of major QTLs and can be used to improve the agronomic and quality traits in cultivated peanut by marker-assisted selection.**

**Abstract:**

Cultivated peanut (*Arachis hypogaea* L.) is an allotetraploid species (AABB, 2*n* = 4× = 40), valued for its edible oil and digestible protein. Linkage mapping has been successfully conducted for most crops, and it has been applied to detect the quantitative trait loci (QTLs) of biotic and abiotic traits in peanut. However, the genetic basis of agronomic and quality-related traits remains unclear. In this study, high levels of phenotypic variation, broad-sense heritability and significant correlations were observed for agronomic and quality-related traits in an *F*_2:3_ population. A genetic linkage map was constructed for cultivated peanut containing 470 simple sequence repeat (SSR) loci, with a total length of 1877.3 cM and average distance of 4.0 cM between flanking markers. For 10 agronomic traits, 24 QTLs were identified and each QTL explained 1.69–18.70 % of the phenotypic variance. For 8 quality-related traits, 12 QTLs were identified that explained 1.72–20.20 % of the phenotypic variance. Several QTLs for multiple traits were overlapped, reflecting the phenotypic correlation between these traits. The majority of QTLs exhibited obvious dominance or over-dominance effects on agronomic and quality traits, highlighting the importance of heterosis for breeding. A comparative analysis revealed genomic duplication and arrangement of peanut genome, which aids the assembly of scaffolds in genomic sequencing of *Arachis* *hypogaea*. Our QTL analysis results enabled us to clearly understand the genetic base of agronomic and quality traits in cultivated peanut, further accelerating the progress of map-based cloning of major QTLs and marker-assisted selection in future breeding.

**Electronic supplementary material:**

The online version of this article (doi:10.1007/s00122-015-2493-1) contains supplementary material, which is available to authorized users.

## Introduction

Cultivated peanut (*Arachis hypogaea* L.) is an allotetraploid (AABB, 2*n* = 4× = 40) species that may be the product of a single hybridization event between *A. duranensis* (AA, 2*n* = 2× = 20) and *A. ipaënsis* (BB, 2*n* = 2× = 20) followed by chromosome duplication (Kochert et al. 1996). Peanut is not only used as human food and an edible oil but also as livestock fodder and green manure. In addition, peanut is cultivated in tropical and sub-tropical regions in more than 100 countries, with a global annual production of 38.6 Mt over an area of 21.8 Mha (http://faostat.fao.org/faostat/collections?subset=agriculture 2011). However, with the rapid increase of human populations worldwide, production has been unable to satisfy global demand. In China, the field area of 4.7 Mha was harvested with a total peanut production of 16.1 Mt, which is the largest area of production worldwide but not the highest yield per hectare (3.4 t ha^−1^), suggesting that there is great potential to further enhance peanut production through the genetic improvement of high-yield varieties.

Yield is one of the most important and complex traits in crops. Peanut yield is directly and indirectly influenced by agronomic traits, such as the height of the main stem and total branching number. Because the pod is one of the most important organs, pods play a paramount role in growth and development processes and serve to protect the developing seeds from biotic and abiotic stresses. Pod- and seed-related traits, such as pod length, pod width, seed length and seed width directly influence peanut yield. In addition, more than 60 % of the total peanut production in China is crushed for edible oil. Fatty acid composition influences the quality and storage stability of the total oil. Thus, oil content and fatty acid composition have also become increasingly important trait objectives in the breeding of high-yield peanut varieties.

Agronomic and quality-related traits are almost always quantitative traits in plant species. Linkage mapping or QTL mapping based on segregating populations derived from bi-parental crossing is the most popular and successful method of identifying QTLs that control complex traits in model plants (Miura et al. 2011; Xing and Zhang 2010). In non-model species, however, QTL mapping is hindered because the number of markers is not large enough to construct saturated genetic maps. SSR markers that feature codominant heritability, genome-wide dispersal and simple amplification have become the most valuable markers for diverse studies including genetic mapping, comparative genomics, molecular fingerprinting and marker-assisted selection (Gupta and Varshney 2000). The first SSR-based genetic linkage map for cultivated peanut was constructed by Varshney et al. (2009) with a recombinant inbred line (RIL) population derived from TAG24 × ICGV86031. Because of low genetic polymorphism between any two varieties of cultivated peanut, only 135 SSR loci were included on the genetic map and classified into 22 linkage groups (LGs). With abundant SSRs developed from various resources, including cDNA libraries (Koilkonda et al. 2012; Proite et al. 2007), SSR-enriched genomic DNA libraries (Naito et al. 2008), bacterial artificial chromosome (BAC)-end sequences (Wang et al. 2012) and transcript sequences (Zhang et al. 2012), a number of genetic linkage maps have been constructed based on *F*_2_ and RIL populations (Gautami et al. 2012a; Qin et al. 2012; Shirasawa et al. 2012; Wang et al. 2012). A high-density genetic linkage map has recently been reported. The map is composed of 16 single-linkage maps and contains 3693 marker loci covering 2651 cM across 20 consensus LGs that include A and B genomes (Shirasawa et al. 2013). Furthermore, a number of linkage studies have been undertaken to identify QTLs for a wide range of various traits in peanut, including resistance to biotic stresses (Khedikar et al. 2010; Leal-Bertioli et al. 2009; Qin et al. 2012; Sujay et al. 2012) and abiotic stress (drought tolerance) (Gautami et al. 2012b; Ravi et al. 2011; Varshney et al. 2009), nutritional quality (protein content, oil content, and oleic acid) (Sarvamangala et al. 2011) and several agronomic traits (Shirasawa et al. 2012). Although great progress has been made for these traits, the QTL pattern of agronomic and quality-related traits remains unclear.

In this study, we developed a molecular genetic map of peanut based on an *F*_2_ mapping population using published SSR markers. By linking with phenotypes across multiple environments, QTL mapping was performed to unravel the genetic basis of 18 agronomic and quality-related traits in peanut.

## Materials and methods

### Plant materials

An *F*_2_ mapping population derived from Zhonghua 10 × ICG12625 (*n* = 232), was used to construct a linkage map. The female parent, Zhonghua 10, belongs to *A. hypogaea* var. *vulgaris* and is large-seeded with pink testa and two seeds per pod. The male parent, ICG12625, belongs to *A. hypogaea* var. *aequatoriana* and is small-seeded with dark purple testa and three seeds in each pod.

### Trait measurement

Because of the low fertility of self-pollinated offspring, only 144 lines in the *F*_3_ generation were obtained. The 144 *F*_3_ lines and parental lines were planted in experimental fields of Wuhan, Nanchong and Zhumadian in China in 2011. Each accession was planted in a single-row plot with the one-replication randomized block design, and there were 8–10 plants in each row with 10 cm between plants within each row and 30 cm between the rows. Eight plants were selected randomly from each accession to investigate the phenotypes. Agronomic traits were investigated according to previously described standard procedures (Jiang et al. 2006), including height of main stem, total branching number, pod length, pod width, seed length, seed width, hundred-pod weight, hundred-seed weight and shelling percentage. The oil content and composition of fatty acids were tested with nuclear magnetic resonance and gas chromatography, respectively, using fresh-dried mature seeds with intact testa. To reduce the influence of environmental factors on phenotypic characterization, the mean value of the phenotypes across three environments for each trait was used in following analyses. A principal component analysis (PCA) of the RIL for the traits and the correlation analysis between the traits and inferred PCs (principal components) were performed using the R package (R Development Core Team 2012).

### DNA extraction and SSR genotyping

Genomic DNA was extracted from young leaves collected from each line using a modified cetyltrimethyl ammonium bromide (CTAB) method. The integrity and quality of the DNA was evaluated on a 1 % agarose gel by comparison with uncut lambda DNA.

A total of 3371 SSR markers from different resources was used to screen the polymorphism between two parental genotypes (Table S1) and polymorphic SSRs were used to genotype the *F*_2_ population. SSR markers with the prefixes pPGPseq, pPGSseq, TC, IPAHM, Ah, RI, EE, EM, GA, GM, GNB, AC, Ad, ARS, gi, AHBGS, PM, AHS, AHGS and HAS were obtained from the literatures (Bravo et al. 2006; Cuc et al. 2008; Ferguson et al. 2004; Gimenes et al. 2007; He et al. 2003; Hopkins et al. 1999; Liang et al. 2009; Macedo et al. 2012; Moretzsohn et al. 2005, 2009; Qin et al. 2012; Shirasawa et al. 2012; Wang et al. 2012; Zhang et al. 2012). SSR markers with prefixes XY and POCR were developed and published by our laboratory (Tang et al. 2012), and those with prefixes AGGS were developed and unpublished by our laboratory. Based on the origin of the SSRs, we classified the total published SSRs (3371) into four groups: genomic SSRs (1467), EST-SSRs (1589), BAC-end SSRs (155), and transcript-SSRs (160). The PCR reactions followed the protocol described by Chen et al. (2008), and the PCR products were visualized on a 6 % polyacrylamide gel followed by silver staining. The fragment sizes of the PCR products were estimated by comparison with a 50 bp DNA ladder.

### Construction of the genetic linkage map

Pearson’s Chi square test was used to assess the goodness of fit to the expected 1:2:1 segregation ratio for each codominant marker or expected 3:1 segregation ratio for each dominant marker (*P* < 0.05). A genetic linkage map was constructed using JoinMap 3.0 (Van and Voorrips 2001) with the minimum logarithm of odds (LOD) of 4.0. The recombination ratio was converted to genetic distance by the Kosambi mapping function (Kosambi 1944). Linkage groups were aligned to a published linkage map based on common markers (Shirasawa et al. 2013), and the linkage groups belonging to the A and B genome were designated A1–A10 and B1–B10, respectively.

### QTL analysis

QTL analyses for agronomic traits, oil content and fatty acids were performed separately. PC significantly related to agronomic traits and quality-related traits were also subjected to QTL analysis. The composite interal mapping method (Zeng 1994) implemented in the software windows QTL cartographer 2.5 (http://statgen.ncsu.edu/qtlcart/WQTLCart.htm) was used to conduct the QTL analysis. The forward regression method model 6 (default model) was selected to obtain covariates. The number of control markers, window size and walk space were set to 5, 10 and 2 cM, respectively. The threshold of LOD for declaring the presence of a QTL was determined by 1000 permutation tests. The nomenclature of QTL was similar to that described by Udall et al. (2006). QTLs are designated with initial letter *q* followed by the trait name and linkage group. An alphabetical letter was added if more than one QTL was detected for the same linkage group for one trait. For example, if two QTLs for seed width were detected on A2, they were named *qSWA2.1* and *qSWA2.2*. Dominance degree (*d*) is defined as the absolute value of dominance divided by additive effect, which was used to classify the detected QTLs for all traits into the following three groups: (1) additive QTL, where 0 < *d* < 0.5; (2) dominance QTL, where 0.5 < *d* < 1; and (3) over-dominance QTL, where *d* > 1 (Zhou et al. 2012).

## Results

### Phenotypic variation of agronomic and quality-related traits

**Fig. 1:**
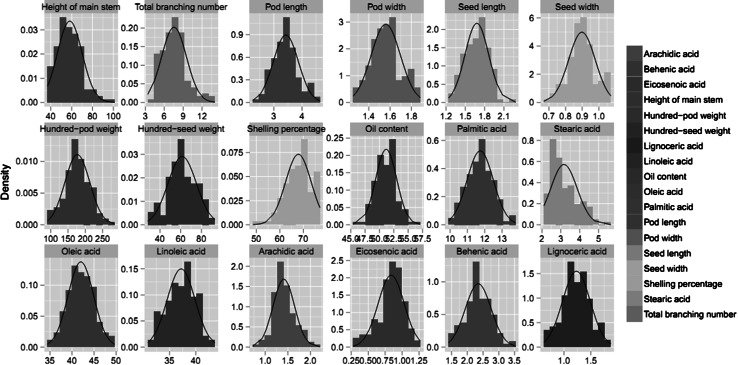
Phenotypic distribution of 18 agronomic and quality traits in the *F*_2:3_ population

Eighteen agronomic and quality-related traits were investigated in the 144 *F*_2:3_ lines in three locations in 2011. An analysis of variance (ANOVA) indicated that the genotypic effect (G) and environmental effect (E) had significant influences on agronomic and quality-related traits (Table 1). Therefore, the mean value of each trait across three environments was used in the subsequent analysis to reduce the instability of phenotypic characterization. Large variation was observed in the *F*_3_ population for these traits (Table 1). Eicosenoic acid showed the largest change (6.0-fold), which varied from 0.3 to 1.8 % and averaged 0.8 %, whereas the oil content showed the lowest change (1.3-fold), which varied from 45.4 to 57.1 % and averaged 51.2 %. A similar phenomenon was observed for the coefficient of variation (CV), which ranged from 3.7 % for the oil content to 30.3 % for the total branching number. In addition, the phenotypic distribution for all of the traits revealed near normality (Fig. 1). The broad sense heritability of all traits was relatively high and ranged from 0.711 for the total branching number to 0.863 for eicosenoic acid (Table 1); however, the broad sense heritability for palmitic acid (0.642) was lower, which indicated that genetic factors were predominant in determining the phenotypes of these traits. A correlation analysis was conducted to determine the association between these traits (Table 2); oleic acid had the highest significant negative association with linoleic acid (*r* = −0.885, *P* < 0.001), whereas, the hundred-pod weight had the highest significant positive association with hundred-seed weight (*r* = 0.856, *P* < 0.001). For pod length, pod width, seed length, seed width, hundred-pod weight and hundred-seed weight, positive correlations were revealed between each pair of the six traits. Similarly, positive correlations were also revealed between each pair of the following traits: height of main stem, total branching number and oil content. Furthermore, negative correlations were revealed between each of the former six traits and each of the latter three traits.

**Table 1:** Phenotypic variation for agronomic and quality-related traits in the peanut panel

Trait	Zhonghua 10	ICG12625	Mean ± SD	CV (%)	Range	Change fold	G	E	*H* ^2a^
Height of main stem (cm)	39.4	64.1	59.2 ± 13.5	22.8	30.6–103.6	3.4	**	**	0.811
Total branching number	9.4	4.2	7.6 ± 2.3	30.3	3.4–19.4	5.7	**	**	0.711
Pod length (cm)	3.2	3.5	3.4 ± 0.5	14.7	1.9–4.6	2.4	**	**	0.830
Pod width (cm)	1.6	1.4	1.6 ± 0.1	6.3	1.2–2.0	1.7	**	**	0.777
Seed length (cm)	1.8	1.3	1.7 ± 0.2	11.8	1.0–2.3	2.3	**	**	0.823
Seed width (cm)	1.1	0.7	0.9 ± 0.1	11.1	0.6–1.1	1.8	**	**	0.775
Hundred-pod weight (g)	209.7	131.5	171.5 ± 39.9	23.3	52.5–284.2	5.4	**	**	0.803
Hundred-seed weight (g)	89.8	49.6	59.2 ± 14.9	25.2	28.3–92.8	3.3	**	**	0.820
Shelling percentage (%)	78.6	56.1	66.7 ± 6.9	10.3	41.2–77.0	1.9	**	**	0.754
Oil content (%)	55.3	52.0	51.2 ± 1.9	3.7	45.4–57.1	1.3	**	**	0.715
Palmitic acid (%)	11.6	13.5	11.7 ± 0.8	6.8	9.6–13.8	1.4	**	**	0.642
Stearic acid (%)	4.8	2.6	3.2 ± 0.7	22.0	2.1–5.6	2.7	**	**	0.848
Oleic acid (%)	46.5	37.9	42.0 ± 3.0	7.1	34.5–49.6	1.4	**	**	0.817
Linoleic acid (%)	33.1	40.0	37.2 ± 2.7	7.3	30.0–43.6	1.5	**	**	0.781
Arachidic acid (%)	1.9	1.3	1.4 ± 0.2	16.9	0.7–2.2	3.1	**	**	0.839
Eicosenoic acid (%)	0.6	1.3	0.8 ± 0.2	24.6	0.3–1.8	6.0	**	**	0.863
Behenic acid (%)	2.2	2.7	2.4 ± 0.4	17.5	1.4–3.5	2.5	**	**	0.738
Lignoceric acid (%)	0.9	1.2	1.2 ± 0.3	20.8	0.6–1.9	3.2	**	**	0.815

** Significant at *P* < 0.01 for the effects of genotype (G) and environment (E) on the phenotypic variance estimated by one-way ANOVA

^a^Family mean-based broad-sense heritability

**Table 2:** Correlation analysis for the agronomic and quality-related traits

Correlation	HMS	TBN	PL	PW	SL	SW	HPW	HSW	SP	OC	PA	SA	OA	LinA	AA	EA	BA	LigA
HMS		0.008	−0.107	0.013	−0.337	−0.436	−0.242	−0.364	−0.462	0.245	0.177	−0.314	−0.146	0.217	−0.162	0.077	0.064	−0.058
TBN	ns		−0.017	0.037	−0.164	−0.165	−0.141	−0.077	−0.240	0.058	0.107	−0.018	−0.161	0.139	−0.006	−0.065	−0.007	0.041
PL	ns	ns		0.414	0.786	0.237	0.636	0.569	0.079	−0.247	−0.047	−0.101	0.023	0.087	−0.198	−0.079	−0.237	−0.072
PW	ns	ns	**		0.532	0.492	0.624	0.542	−0.155	−0.297	0.031	−0.050	−0.011	0.059	−0.138	−0.057	−0.113	0.042
SL	**	ns	**	**		0.641	0.832	0.841	0.379	−0.318	−0.144	0.035	0.215	−0.093	−0.185	−0.162	−0.331	−0.044
SW	**	ns	**	**	**		0.726	0.824	0.583	−0.288	−0.103	0.136	0.243	−0.202	−0.085	−0.158	−0.234	0.015
HPW	**	ns	**	**	**	**		0.856	0.405	−0.341	−0.052	−0.045	0.126	−0.036	−0.201	−0.088	−0.269	−0.004
HSW	**	ns	**	**	**	**	**		0.520	−0.416	−0.131	0.058	0.214	−0.145	−0.140	−0.148	−0.253	0.061
SP	**	**	ns	ns	**	**	**	**		−0.138	−0.143	0.190	0.250	−0.258	−0.023	−0.186	−0.212	−0.055
OC	**	ns	**	**	**	**	**	**	ns		0.021	0.003	−0.072	0.087	0.110	0.026	0.087	−0.071
PA	*	ns	ns	ns	ns	ns	ns	ns	ns	ns		−0.507	−0.503	0.486	−0.496	0.230	−0.127	−0.121
SA	**	ns	ns	ns	ns	ns	ns	ns	*	ns	**		0.146	−0.276	0.707	−0.554	0.129	−0.109
OA	ns	ns	ns	ns	*	**	ns	*	**	ns	**	ns		−0.885	0.003	−0.222	−0.273	−0.161
LinA	*	ns	ns	ns	ns	*	ns	ns	**	ns	**	**	**		−0.227	0.157	0.084	−0.029
AA	ns	ns	*	ns	*	ns	*	ns	ns	ns	**	**	ns	**		−0.164	0.543	0.328
EA	ns	ns	ns	ns	ns	ns	ns	ns	*	ns	**	**	*	ns	ns		0.258	0.433
BA	ns	ns	**	ns	**	**	**	**	*	ns	ns	ns	**	ns	**	**		0.633
LigA	ns	ns	ns	ns	ns	ns	ns	ns	ns	ns	ns	ns	ns	ns	**	**	**	

*HMS* height of main stem, *TBN* total branching number, *PL* pod length, *PW* pod width, *SL* seed length, *SW* seed width, *HPW* hundred-pod weight, *HSW* hundred-seed weight, *SP* shelling percentage, *OC* oil content, *PA* palmitic acid, *SA* stearic acid, *OA* oleic acid, *LinA* linoleic acid, *AA* arachidic acid, *EA* eicosenoic acid, *BA* behenic acid, *LigA* lignoceric acid

** Significant at *P* < 0.01; * significant at *P* < 0.05; *ns* non-significant at *P* < 0.05

### SSR polymorphisms and genetic map construction

From a total of 3371 SSR markers, a set of 341 Genomic SSRs (23.24 %), 82 EST-SSRs (5.16 %), 32 BAC-end SSRs (20.65 %) and 15 transcript-SSRs (9.38 %) were polymorphic between the parents ‘Zhonghua 10’ and ‘ICG12625’, indicating that random genomic SSRs had higher polymorphism than SSRs within coding regions. Four hundred-seventy polymorphic SSR primers were used to genotype the *F*_2_ population of 232 individuals (Table 3). According to the SSR band segregation patterns in the *F*_2_ population, 26 of 470 SSRs amplified two loci for each SSR, and the remaining 444 SSRs amplified a single locus for each SSR. Among these 496 SSRs, 461 SSRs were co-dominant loci and 35 were dominant.

**Table 3:** Polymorphic ratio of the investigated markers

Marker types	Genomic SSR	EST-SSR	BAC-SSR	Transcript-SSR	Total
No. of tested markers	1467	1589	155	160	3371
No. of polymorphic markers between parent materials	341	82	32	15	470
Polymorphism percentage (%)	23.24	5.16	20.65	9.38	13.94

**Fig. 2:**
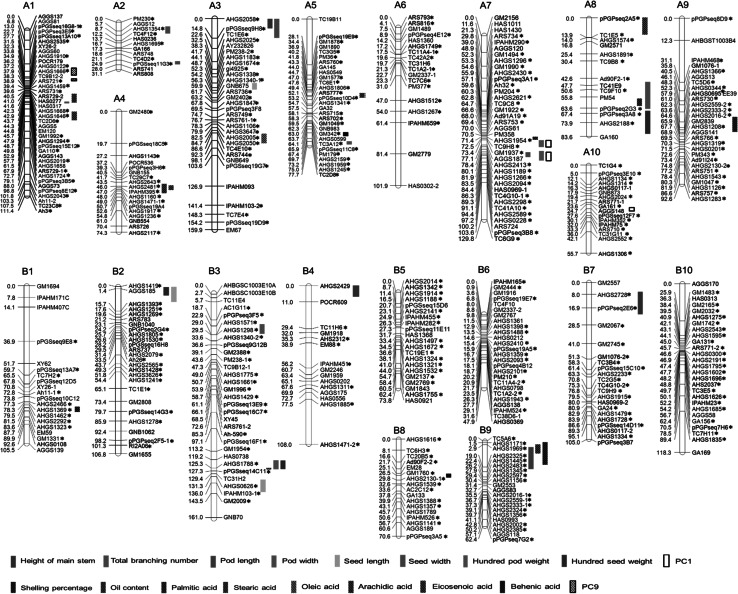
Genetic linkage map and QTL distribution of the agronomic and quality-related traits in an *F*_2:3_ population derived from Zhonghua 10 × ICG12625. The suffix “*asterisk*” indicates loci with segregation distortion in *F*_2_ generation, and the *red loci* are common loci for assigning linkage groups to defined chromosomes of cultivated peanut based on a comparison with the published map (color figure online)

A genetic linkage map containing 470 SSR loci was constructed, and it covered a total length of 1877.3 cM of the cultivated peanut genome, with an average distance of 4.0 cM between flanking markers (Fig. 2; Table 4). All of the loci were assigned to 20 linkage groups (LG) that were designated as A1–A10 for the A subgenome and B1–B10 for the B subgenome by aligning common markers to previously published maps. Out of 470 mapped SSR loci, the Chi square (*χ* ^2^) analysis identified 133 loci (28.3 %) that significantly deviated from expected ratios of 1:2:1 or 3:1 (*P* < 0.05). The number of segregation-distorted loci varied for the different linkage groups, ranging from 1 locus (5.0 %) in B1 to 16 loci (61.5 %) in B2 (Fig. 2; Table S2).

**Table 4:** Description of the peanut genetic linkage map in this study

Linkage group	Length (cM)^a^	Number of loci^b^	Segregation distorted loci^c^	Inverted common loci^d^
A1	111.4	40 (25)	14 (0.35)	13 (0.52)
A2	31.1	12 (6)	6 (0.5)	3 (0.5)
A3	159.9	31 (25)	4 (0.13)	13 (0.52)
A4	74.3	18 (13)	4 (0.22)	7 (0.54)
A5	77.7	29 (21)	5 (0.17)	14 (0.67)
A6	101.9	18 (12)	10 (0.56)	6 (0.5)
A7	129.8	37 (28)	7 (0.19)	15 (0.54)
A8	83.6	14 (10)	3 (0.21)	6 (0.6)
A9	92.6	29 (23)	3 (0.1)	13 (0.57)
A10	55.7	17 (13)	8 (0.47)	6 (0.46)
B1	105.5	20 (10)	1 (0.05)	4 (0.4)
B2	106.8	26 (18)	16 (0.62)	11 (0.61)
B3	161	31 (21)	12 (0.39)	10 (0.48)
B4	108	15 (8)	7 (0.47)	3 (0.43)
B5	73.8	21 (16)	2 (0.1)	5 (0.31)
B6	47.9	26 (15)	5 (0.19)	8 (0.53)
B7	105	21 (17)	9 (0.43)	6 (0.35)
B8	70.6	17 (13)	1 (0.06)	4 (0.31)
B9	62.4	21 (17)	2 (0.1)	11 (0.65)
B10	118.3	27 (23)	14 (0.52)	13 (0.57)
A sub-genome	918	245 (176)	64 (0.26)	96 (0.55)
B sub-genome	959.3	225 (157)	69 (0.31)	75 (0.48)
Whole genome	1877.3	470 (333)	133 (0.28)	171 (0.51)

^a^Genetic length of each chromosome, sub-genome and whole genome

^b^SSR loci mapped on the linkage map and common loci mapped on the linkage map (in parentheses)

^c^Number of SSR loci with segregation distortion and proportions of mapped loci (in parenthesis) in *F*_2_ generation

^d^Number of common SSR loci that are not co-linear with the published linkage map and proportions of common loci (in parentheses)

### Comparative analyses of the inter-genome and intra-genome

**Fig. 3:**
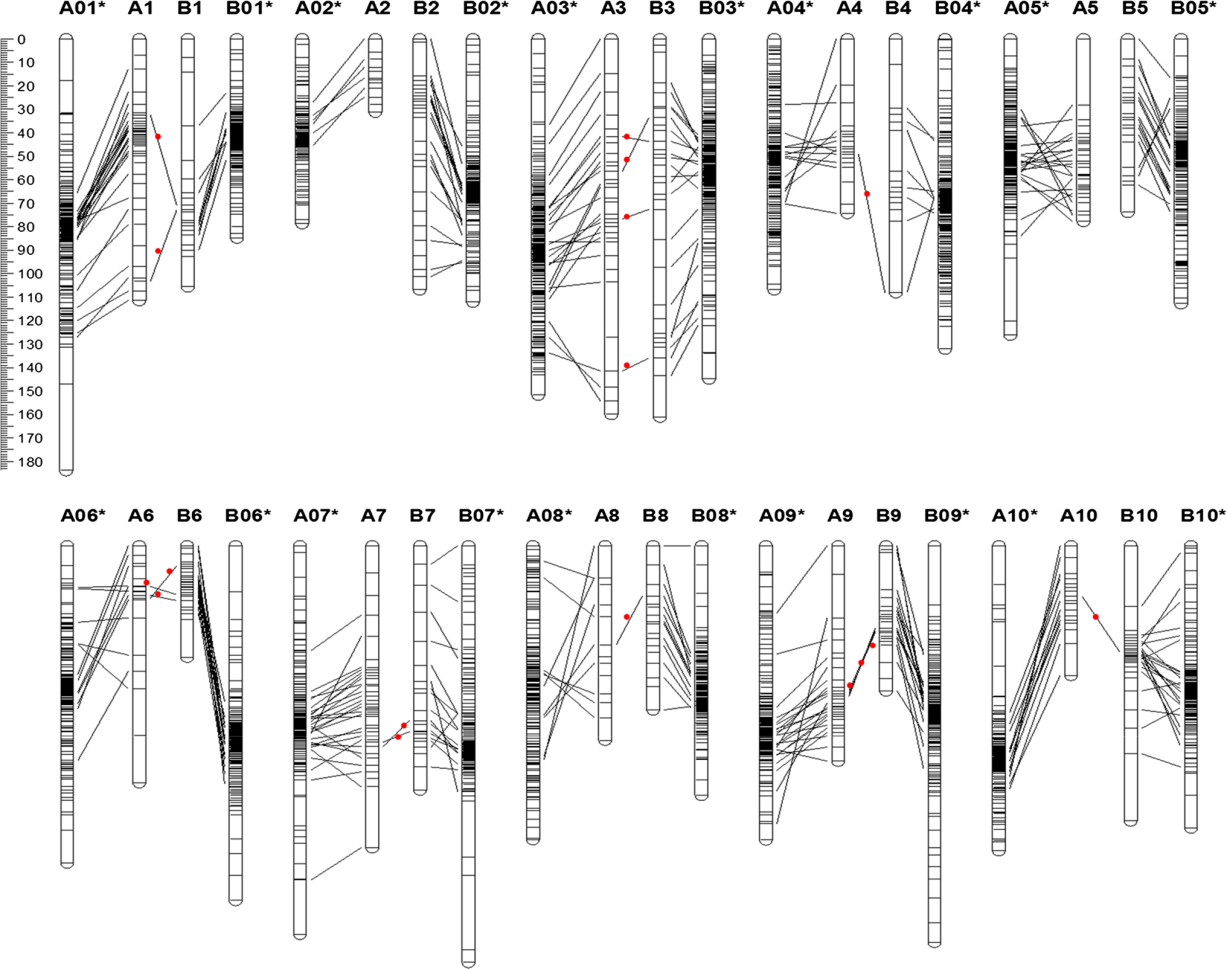
Comparison of the linkage map with the published linkage map for cultivated peanut (Shirasawa et al. 2013). The suffix “*asterisk*” indicates the linkage group on the published linkage map. Co-linear SSR loci between two linkage maps and homoeologous loci between the A and B genomes are indicated by *black lines*. *Red dots* highlights the homoeologous relationships between subgenomes (color figure online)

The syntenic comparative analysis of the present linkage map with the published tetraploid linkage map (Shirasawa et al. 2013) revealed distinct similarities. Of the 470 mapped SSRs in the present linkage map, 333 SSRs (70.9 %) were common loci with the published linkage map, ranging from 6 common loci for A2 to 28 common loci for A7 (Table 4). A small fraction of inverted segments, which were defined as consecutively inverted common loci, were observed in the comparison analysis, indicating that our linkage map is essentially co-linear to the published linkage map (Fig. 3; Table 4).

Analyzing the duplicated loci amplified from the same SSR primer pair is a direct method of determining the homology in allotetraploid genomes. We detected 26 homologous SSRs that amplified 52 duplicated loci. Among the 26 homologous SSRs, 4 singleton SSRs were detected where only one homologous locus was mapped on the linkage map, indicating that the detection of homologous loci was highly likely, whereas the detection of allelic polymorphisms was not as likely. Moreover, 17 homoeologus SSRs (65.4 %) amplified two duplicated loci for each SSR and were mapped onto the A subgenome and B subgenome (Fig. 3). For example, SSR primer Ah11 amplified two loci, Ah11-1 and Ah11-2, which were mapped on chromosomes B1 and A1, respectively. The SSR primer pair AHGS2130 amplified two loci, AHGS2130-1 and AHGS2130-2, which were mapped on chromosome B8 and A9, respectively. Additionally, only one SSR showed a potentially homologous relationship within subgenomes. The SSR primer ARS729 amplified two loci ARS729-1 and ARS729-2, which were both mapped on the chromosome A1, suggesting the two loci may be originated from a local duplication event during genome evolution of *A. duranensis*.

### Detection of QTLs for agronomic and quality-related traits

Genome-wide QTL mapping was performed using the mean value of each trait across three environments (Fig. 2; Table 5). For the agronomic traits, a total of 24 QTLs were identified that explained 1.69–18.70 % of the phenotypic variance. Three QTLs for the height of main stem, *qHMSA3*, *qHMSB4* and *qHMSB7*, were detected on A3, B4 and B7, and they explained 8.31, 6.12 and 8.90 % of the phenotypic variance, respectively. Two QTLs for total branching number, *qTBNA1* and *qTBNA8*, were detected on A1 and A8, and they explained 7.46 and 6.11 % of the phenotypic variance, respectively. One QTL for pod length, *qPLB9*, was detected on B9, and it explained 11.23 % of the phenotypic variance. Two QTLs for pod width, *qPWA5* and *qPWB3*, were detected on A5 and B3, and they explained 18.70 % and 2.11 % of the phenotypic variance, respectively. Three QTLs for seed length, *qSLA3*, *qSLB2* and *qSLB3*, were detected, and explained 9.86–10.48 % of the phenotypic variance. Four QTLs for seed width, *qSWA2.1*, *qSWA2.2*, *qSWA3* and *qSWA7*, were detected and explained 6.39–12.20 % of the phenotypic variance. Three QTLs for hundred-pod weight, *qHPWA7.1*, *qHPWA7.2* and *qHPWB3*, were detected and explained 8.02–15.39 % of the phenotypic variance. Three QTLs for hundred-seed weight, *qHSWA8*, *qHSWB2* and *qHSWB3*, were detected and explained 1.69–17.88 % of the phenotypic variance. Three QTLs for shelling percentage, *qSPA5*, *qSPA7* and *qSPA9*, were detected and explained 2.00–11.78 % of the phenotypic variance.

**Table 5:** QTL for the agronomic and quality-related traits

Trait^a^	QTL^b^	Linkage group	LOD	Pos. (cM)	*R* ^2c^	Interval (cM)^d^	*A* ^e^	*D* ^f^	*d* ^g^
HMS	***qHMSA3***	A3	2.9	0.01	8.31	0–8.1	−5.230	0.269	0.05
	*qHMSB4*	B4	2.5	0.01	6.12	0–8.6	−1.809	6.458	3.57
	*qHMSB7*	B7	2.8	16.91	8.90	5.8–22.6	4.799	−1.269	0.26
TBN	*qTBNA1*	A1	2.8	48.81	7.46	48.5–49.8	−0.251	1.117	4.45
	***qTBNA8***	A8	3.7	47.81	6.11	45.4–64	−0.090	1.515	16.80
PL	***qPLB9***	B9	2.6	16.91	11.23	5.3–20.7	0.139	−0.170	1.22
PW	*qPWA5*	A5	2.7	76.01	18.70	75–77	0.063	−0.061	0.97
	*qPWB3*	B3	2.6	25.91	2.11	22.3–29	−0.002	0.097	60.56
SL	*qSLA3*	A3	2.6	52.31	10.48	47–54.1	0.059	−0.056	0.94
	***qSLB2***	B2	2.8	0.01	9.86	0–10.7	0.037	−0.097	2.62
	*qSLB3*	B3	2.7	137.01	10.41	133.6–142.8	−0.051	0.083	1.61
SW	*qSWA2.1*	A2	3.3	5.81	8.55	2.6–7.5	0.014	−0.051	3.73
	*qSWA2.2*	A2	3.6	26.91	6.39	25.1–28.1	−0.011	−0.065	5.95
	***qSWA3***	A3	2.6	7.01	12.20	0–14	0.028	−0.036	1.29
	***qSWA7***	A7	2.7	83.51	11.62	82.2–85.4	−0.028	0.033	1.18
HPW	*qHPWA7.1*	A7	3.3	78.11	14.33	74.1–79.4	−17.068	9.978	0.58
	***qHPWA7.2***	A7	3.8	86.31	15.39	81.8–88.3	−15.140	14.243	0.94
	***qHPWB3***	B3	2.7	125.31	8.02	120.6–126.3	−5.199	20.897	4.01
HSW	*qHSWA8*	A8	2.7	75.91	1.69	65.9–81.9	1.766	9.317	5.28
	***qHSWB2***	B2	3.7	0.01	17.88	0−5.3	6.029	−6.566	1.09
	***qHSWB3***	B3	3.5	124.21	11.51	120.5–126	−2.424	9.606	3.96
SP	*qSPA5*	A5	3.0	69.71	6.08	69.3–72.7	−0.269	4.339	16.15
	*qSPA7*	A7	3.0	72.51	11.78	71.9–73.2	−2.581	1.755	0.68
	*qSPA9*	A9	2.7	59.01	2.00	55.3–63.4	2.414	1.758	0.73
OC	***qOCB3***	B3	3.9	126.31	14.36	125.3−126.7	0.312	−1.773	5.68
PA	***qPAA8***	A8	3.0	59.91	17.02	57.1–62	−0.527	−0.026	0.05
OA	*qOAA3*	A3	2.8	91.61	1.72	86.6–94.1	0.368	2.207	6.01
EA	***qEAA1.1***	A1	3.1	47.91	3.80	42.4–48.8	−0.015	−0.138	9.15
	*qEAA1.2*	A1	2.9	59.71	6.04	55–60.7	0.003	−0.157	62.68
	*qEAA5*	A5	2.6	47.01	7.51	46.4–48.8	−0.022	0.118	5.30
BA	*qBAB1*	B1	3.9	81.51	15.76	80.8–84.8	−0.134	0.237	1.78
	***qBAB9***	B9	3.4	9.91	18.85	1.5–16.6	−0.174	0.202	1.16
AA	***qAAA4***	A4	3.7	47.01	8.10	44.4–50.6	0.028	−0.151	5.46
	***qAAB9***	B9	4.8	3.91	20.20	2.6–9.4	−0.131	0.052	0.39
SA	***qSAA4***	A4	3.3	48.01	18.31	45.5–48.8	0.333	−0.270	0.81
	*qSAA8*	A8	3.1	3.01	2.52	0–10.2	−0.048	−0.517	10.89
PC1	***qPC1A7.1***	A7	2.9	78.11	12.80	74.2–79.4	16.714	−9.956	0.60
	***qPC1A7.2***	A7	3.4	86.31	13.86	82.8–88.2	15.817	−12.255	0.77
	*qPC1A10*	A10	2.9	27.61	1.20	26.7–29.2	−17.074	−13.021	0.76
PC9	*qPC9A1.1*	A1	2.7	27.81	5.89	25.1–30.4	0.421	0.060	0.14
	*qPC9A1.2*	A1	2.7	33.31	5.09	32.3–34.4	0.590	0.118	0.20

^a^Abbreviations previously noted; PC1 and PC9 represent the top 2 PCs that are correlated with the agronomic and quality traits, respectively

^b^Italicized, bold and underlined QTLs are overlapped between multiple traits

^c^Phenotypic variance explained by each QTL

^d^QTL support interval at *α* < 0.05

^e^Additive effect of QTL

^f^Dominance effect of QTL

^g^Dominance degree, i.e., the absolute values of the dominance divided by the additive effect

For the quality-related traits, 12 QTLs were identified and explained 1.72–20.20 % of the phenotypic variance. One QTL for oil content, *qOCB3*, was detected on B3, and it explained 14.36 % of the phenotypic variance. One QTL for palmitic acid, *qPAA8*, was detected on A8, and it explained 17.02 % of the phenotypic variance. Two QTLs for stearic acid, *qSAA4* and *qSAA8*, were detected on A4 and A8, and they explained 18.31 and 2.52 % of the phenotypic variance, respectively. One QTL for oleic acid, *qOAA3*, was detected on A3, and it explained 1.72 % of the phenotypic variance. Two QTLs for arachidic acid, *qAAA4* and *qAAB9*, were detected on A4 and B9, and they explained 8.10 % and 20.20 % of the phenotypic variance, respectively. Three QTLs for eicosenoic acid, *qEAA1.1*, *qEAA1.2* and *qEAA5*, were detected and explained 3.80–7.51 % of the phenotypic variance. Two QTLs for behenic acid, *qBAB1* and *qBAB9*, were detected on B1 and B9, and they explained 15.76 and 18.85 % of the phenotype variance, respectively.

A PCA was performed to summarize the phenotypic variation of 18 agronomic and quality traits. Two principal components, PC1 and PC9, were significantly correlated with agronomic and quality traits, respectively (Table S3), and designated as the synthetic phenotypes for QTL mapping. Three QTLs for PC1, *qPC1A7.1*, *qPC1A7.2* and *qPC1A10*, were identified, and they explained 1.20–13.86 % of the phenotypic variance. Two QTLs for PC9, *qPC9A1.1* and *qPC9A1.2*, were identified, and they explained 5.89 % and 5.09 % of the phenotypic variance, respectively. Among these five QTLs, *qPC1A7.1* was co-localized with *qHPWA7.1* for hundred-pod weight and *qPC1A7.2* was co-localized with *qSWA7* for seed width and *qHPWA7.2* for hundred-pod weight.

### Genetic effect of QTLs for agronomic and quality traits

**Fig. 4:**
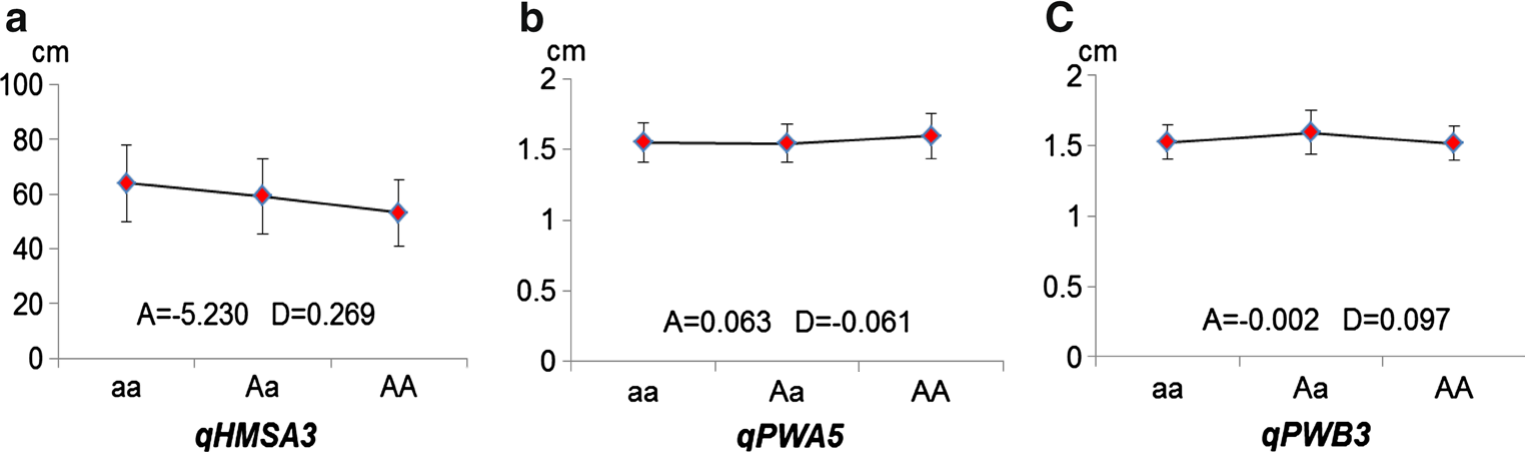
Additive and dominance patterns of three QTLs exhibited differential contributions to phenotypic variance. **a** *qHMSA3* additive QTL with a weak dominance effect relative to the additive effect, **b** *qPWA5* dominance QTL with a dominance effect that is equivalent to the additive effect, **c** *qPWB3* over-dominance QTL with a strong dominance effect relative to the additive effect

The *F*_2:3_ population is an ideal population that can be used to designate QTL genetic effects as either additive or dominant. The degree of dominance may reveal the degree of heterosis for the QTL (Table 5; Fig. 4, S1). Out of 36 QTLs for agronomic and quality traits, four QTLs (11.1 %) exhibited no apparent dominance effect over the additive effect. Seven QTLs (19.4 %) had the mid-parent dominance effect. Twenty-five QTLs (69.4 %) had dominance degree (*d*) that was more than one, exhibiting over-parent heterosis for these QTLs. The results suggested that most of the QTLs for agronomic and quality traits are determined by the dominance effect, which indicates the potential of heterosis applications in peanut breeding.

## Discussion

A genetic linkage map is a prerequisite to efficiently identify molecular markers associated with agronomically important traits. Because of a lack of polymorphism at the DNA level, the first SSR-based genetic linkage map for cultivated peanut, which was constructed by Varshney et al. (2009) only had 135 SSR loci mapped onto 22 linkage groups. As more SSRs have been developed for peanut, more SSR-based maps have been constructed (Qin et al. 2012; Shirasawa et al. 2013, 2012; Sujay et al. 2012; Wang et al. 2012). In the present study, an *F*_2_ mapping population was used to construct a linkage map containing 470 loci, that covered a total length of 1877.3 cM with an average distance of 4.0 cM between adjacent loci. The density of our map was relatively higher than that of other studies, except for the synthetic genetic map which integrated the marker information of 16 linkage maps (Shirasawa et al. 2013). The proportion of SSR loci (29 %) observed to be segregating with distortion was significantly less than previously reported (Moretzsohn et al. 2005; Shirasawa et al. 2013; Wang et al. 2012). Generally, segregation distortion is a result of chromosomal structural differences or the presence of transmission ratio distorter factors on a chromosome (Rieseberg et al. 1995). Therefore, the lower degree of segregation distortion in the present study likely implies a lower degree of diversity in the parental genotypes. Additionally, we observed a lower segregation distortion proportion (19 %) in the 144 RIL lines with the same set of markers (Table S2), indicating the following QTL analysis will be reliable although population size decreased with the *F*_2:3_ population. Interestingly, we found 26 SSR primers for which two duplicated loci in the linkage map exhibited features of homoeologous and paralogues in tetraploid species (Ravi et al. 2011; Varshney et al. 2009). A comparative analysis revealed a small degree of inverted segments between the present linkage map and published integrated map and indicated a high degree of consistency between our linkage map and published maps. Therefore, the linkage map in our study is a beneficial tool that will aid in the assembly of whole genome sequences for tetraploid peanut.

The genetic bases of yield-related traits have been determined in many crop species using QTL mapping (Maccaferri et al. 2008; Xing and Zhang 2010), and several major QTLs have been isolated by map-based cloning (Bommert et al. 2013; Li et al. 2014). In this study, a total of 36 QTLs were detected for 18 agronomic and quality traits. Furthermore, two PCs correlated with agronomic and quality traits were treated as synthetic traits for QTL mapping (Table 5). As a result, five QTLs were detected for the PCs, two of which co-localized with *qHPWA7.1* and *qSWA7* for hundred-pod weight and seed width, respectively. This result indicates that the QTLs of hundred-pod weight and seed width are critically important for determining the phenotypes of agronomic traits in cultivated peanut. Several QTLs for multiple traits co-localized with each other (Table 5), indicating that pleiotropic QTLs or several linked QTLs may be involved in regulating these traits. For example, the QTL *qHPWB3* was detected for hundred-pod weight at the genetic location 120.6–126.3 cM on linkage group B3. Another two QTLs, *qHSWB3* and *qOCB3*, were detected for hundred-seed weight and oil content at the genetic locations of 120.5–126 and 125.3–126.7 cM on linkage group B3, respectively. The co-localization of QTLs for three traits also reflects the phenotypic correlation between these traits (Table 2). For these overlapping QTLs, the allele derived from Zhonghua 10 increased the value of hundred-pod weight and hundred-seed weight but decreased the value of oil content (Table 5). Thus, it is necessary to fine-map these overlapping QTLs to determine causative genes for the overlapped QTLs caused by several linkage QTLs/genes. By utilizing PCR-based molecular markers within or closely linked to the target gene, the potential for effectively pyramiding favorable alleles for multiple traits into single varieties of peanut in selective breeding can be achieved. Furthermore, the majority of QTLs exhibited dominance or over-dominance effects on agronomic and quality traits; thus, determining the heterosis of key agricultural traits should be prioritized in efforts to enhance the elite peanut varieties.

### **Author contribution statement**

LH and HFJ designed the experiment. LH and XPR established the mapping population. HYH and WGC performed SSR genotyping. LH, HYH, WGC, YLC, XJZ, YLX and XLW performed the agronomic traits measurements of the plant materials. XPR performed the composition of fatty acid measurements of the plant materials. XGJ performed the oil content measurements of the plant materials. LH constructed the genetic linkage mapping, performed the QTL analysis and wrote the manuscript. LH, BSL and HFJ revised the manuscript. All of the authors read and approved the final manuscript.

## Electronic supplementary material

Below is the link to the electronic supplementary material.

Supplementary material 1 (PPT 327 kb)

Supplementary material 2 (XLS 101 kb)

Supplementary material 3 (XLS 76 kb)

Supplementary material 4 (XLS 31 kb)

## Abbreviations

ANOVA: Analysis of variance
BAC: Bacterial artificial chromosome
cDNA: Complementary DNA
CIM: Composite interval mapping
CTAB: Cetyltrimethyl ammonium bromide
CV: Coefficient of variation
EST: Expressed sequence tag
LG: Linkage group
LOD: Logarithm of odds
PCA: Principal component analysis
PCR: Polymerase chain reaction
QTL: Quantitative trait locus
SSR: Simple sequence repeat
