# Supplementary figures and images for: Quantitative trait locus analysis of agronomic and quality-related traits in cultivated peanut (Arachis hypogaea L.)
Source: Theor Appl Genet. 2015 Mar 25;128(6):1103–15. doi: 10.1007/s00122-015-2493-1 (PMC4434864; doi:10.1007/s00122-015-2493-1)

## Slide 1
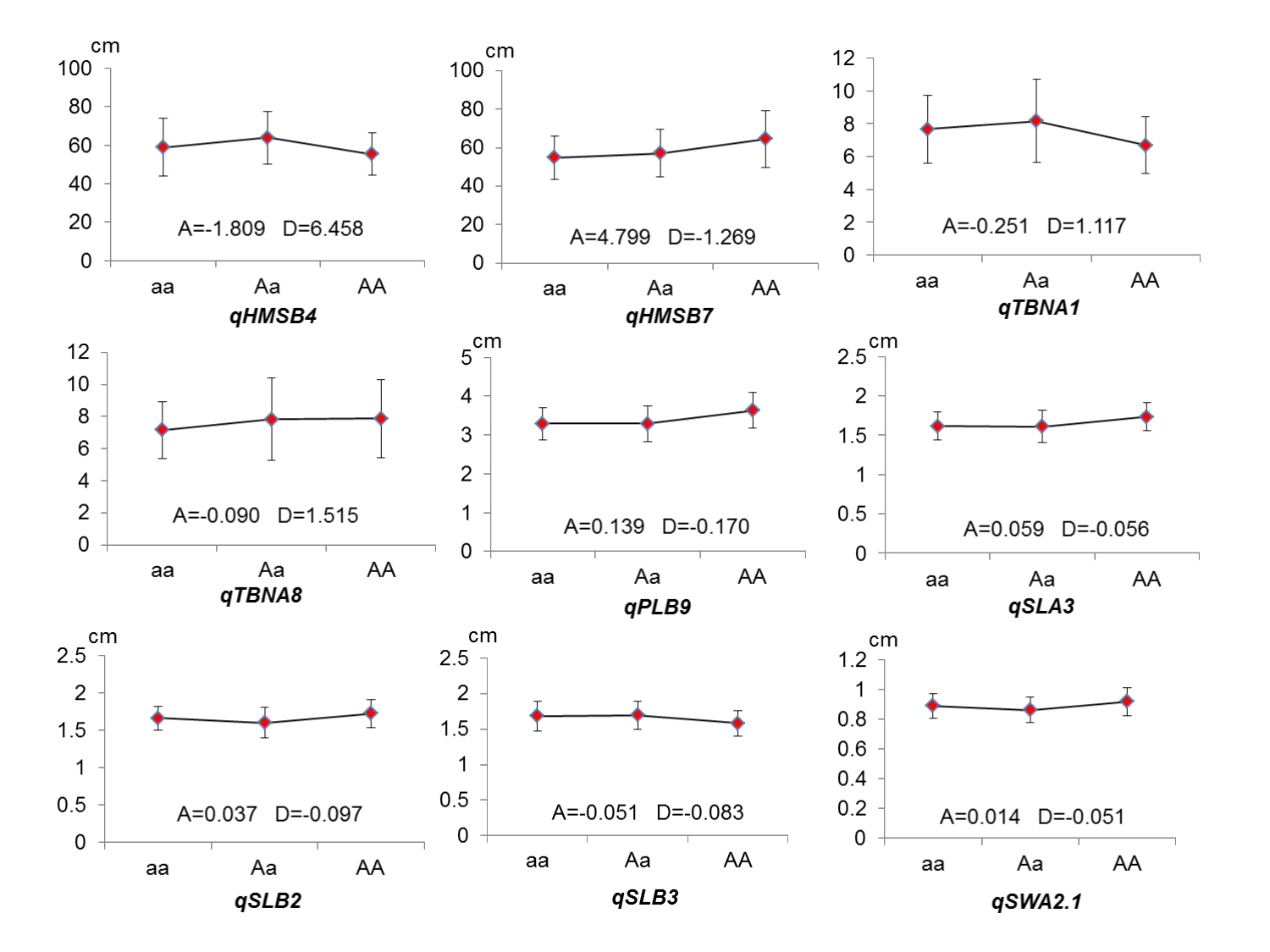

## Slide 2
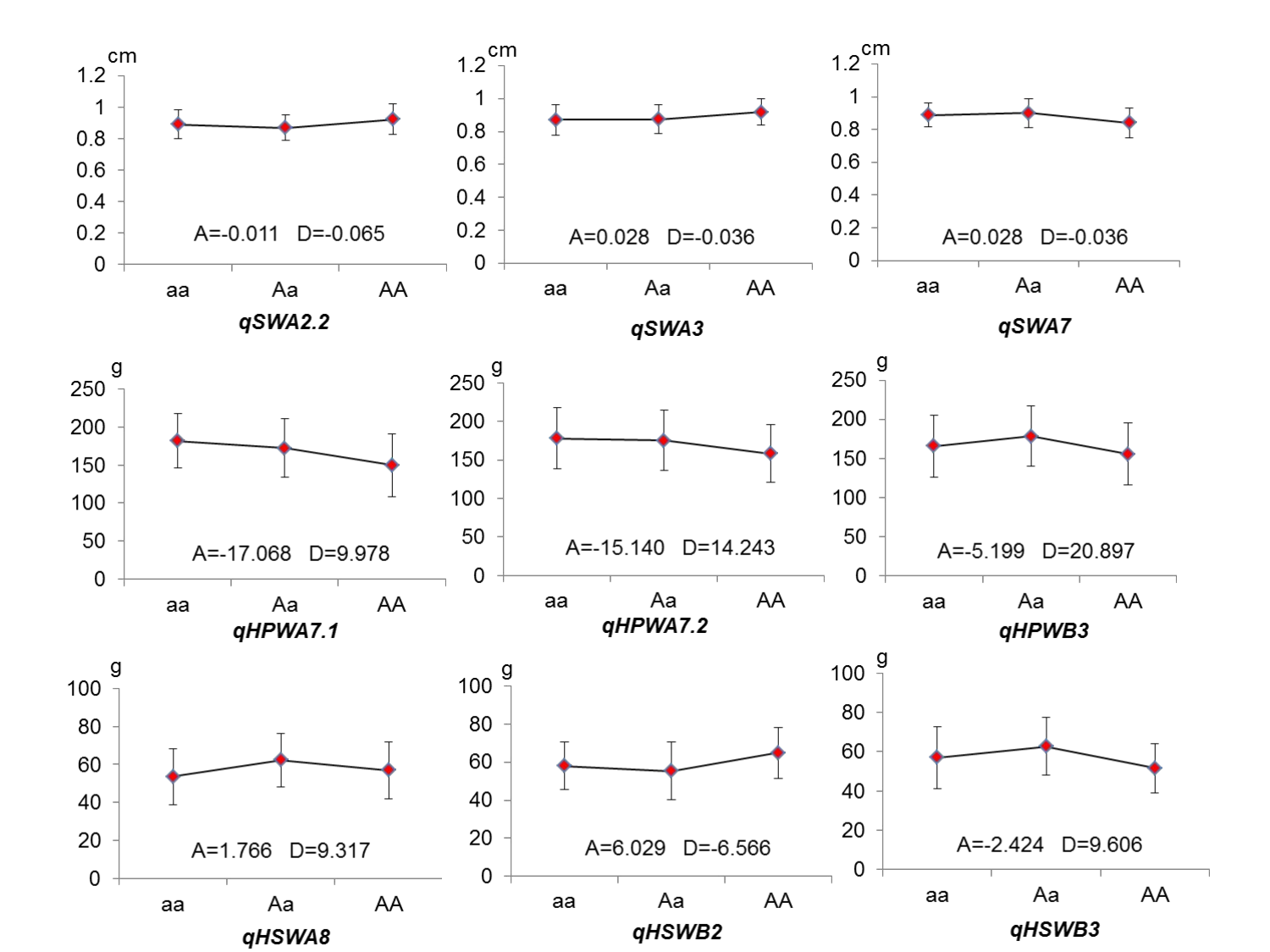

## Slide 3
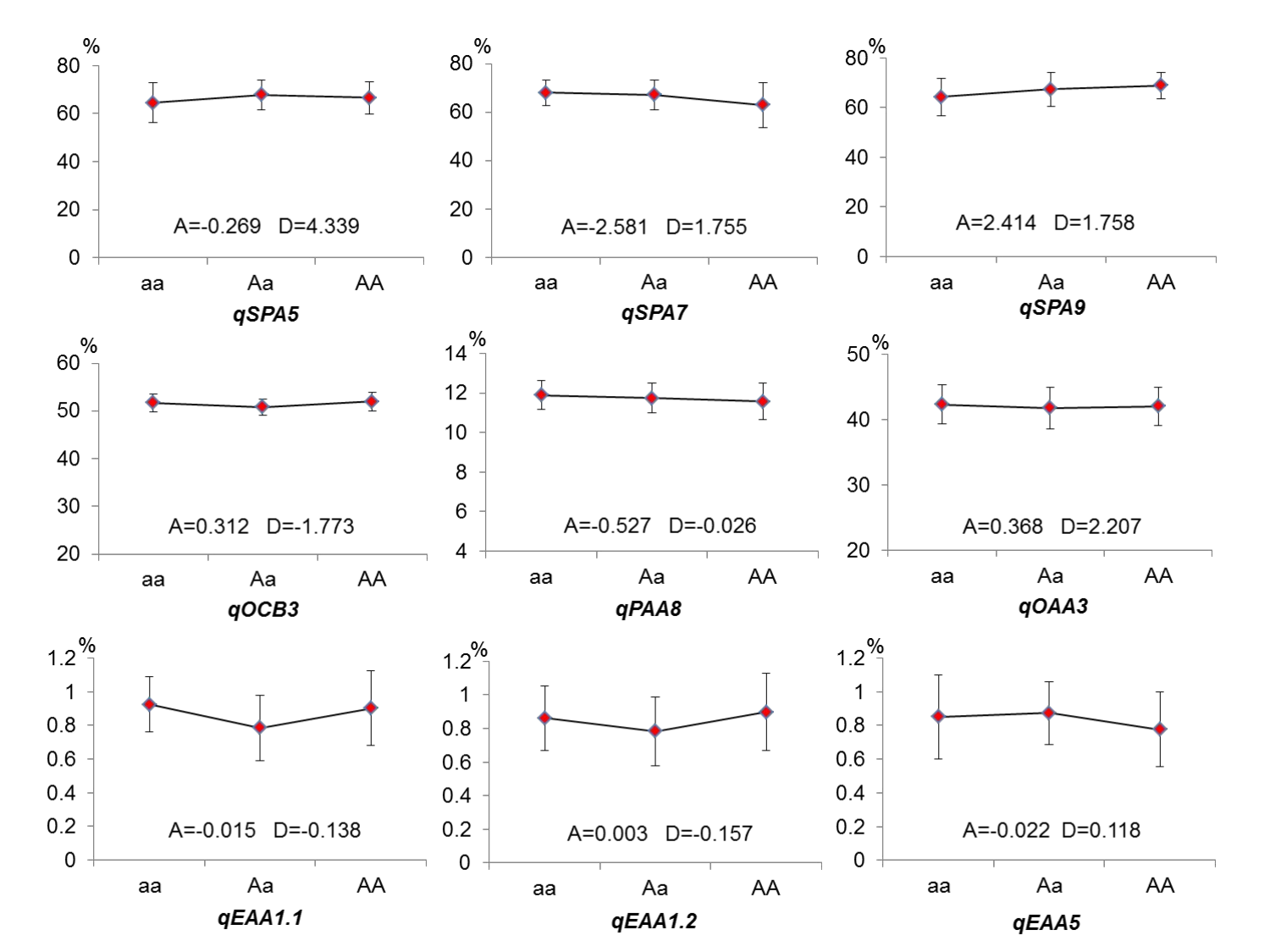

## Slide 4
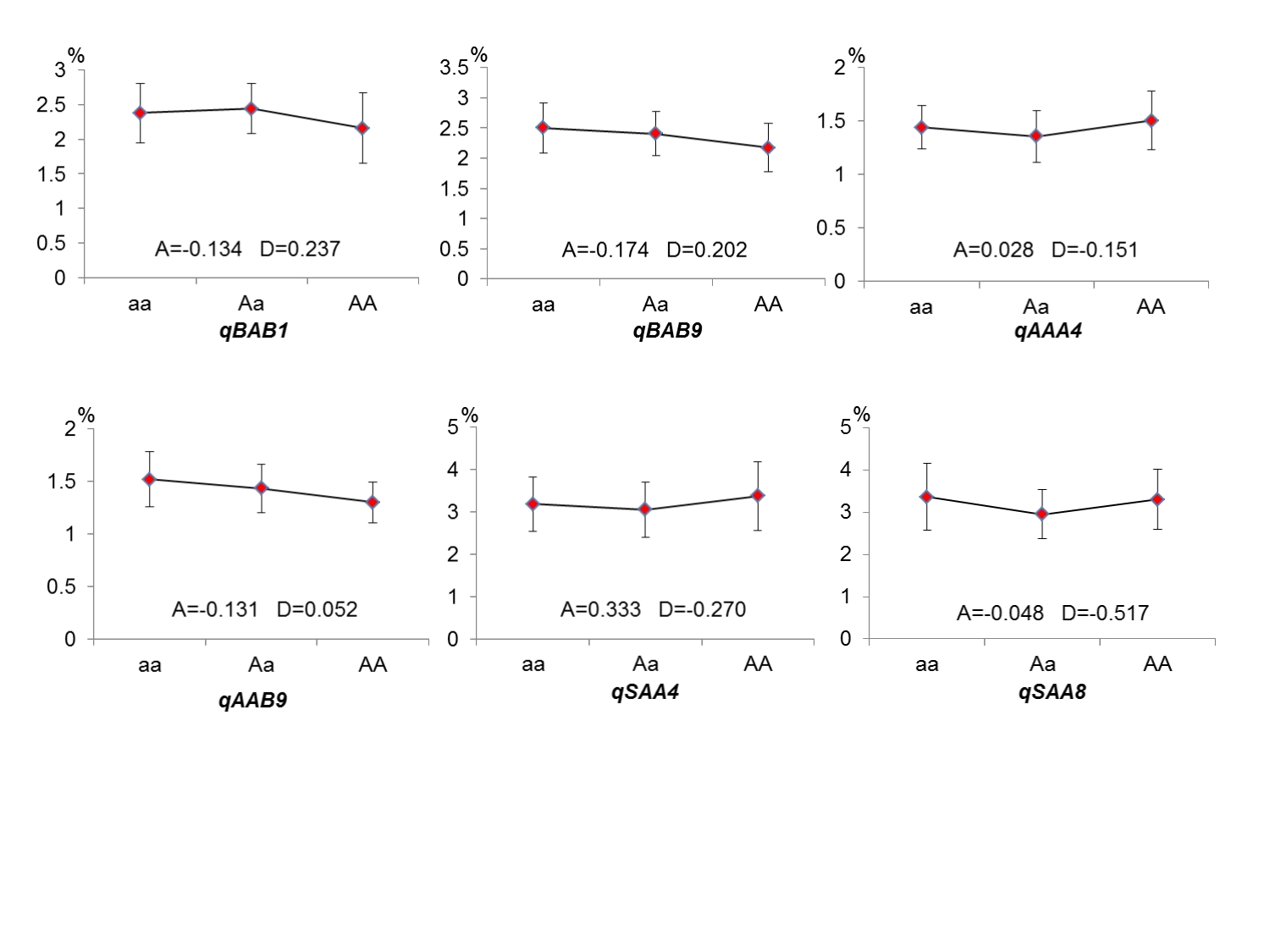

Supplement: Supplementary file 1 — Supplementary material 1 (PPT 327 kb) [file 122_2015_2493_MOESM1_ESM.ppt]
